# Clinical study on treatment of facial seborrheic dermatitis with intense pulsed light combined with 30% supramolecular salicylic acid

**DOI:** 10.6061/clinics/2020/e1875

**Published:** 2020-11-02

**Authors:** Rui-Long Gu, Shan-Qing Wang

**Affiliations:** I Shanghai Huamei Medical Cosmetic Hospital, Shanghai, 200120, China; II College of Aesthetic Medicine, Yichun University, Yichun, 336000, China

**Keywords:** Facial Seborrheic Dermatitis, Intense Pulsed Light, 30% Supramolecular Salicylic Acid, Curative Effect

## Abstract

**OBJECTIVES:**

In this study, we investigated the effects of intense pulsed light (IPL) combined with 30% supramolecular salicylic acid on facial seborrheic dermatitis.

**METHODS:**

A total of 45 patients with mild or moderate facial seborrheic dermatitis were selected from our hospital between September 2018 and September 2019. The patients were divided into three groups consisting of 15 patients each. The first group was exposed to a combination of IPL and 30% supramolecular salicylic acid treatment, the second group was exposed to the IPL treatment alone, and the third group was exposed to the 30% supramolecular salicylic acid treatment alone. They were treated once every 4 weeks in three consecutive rounds.

**RESULTS:**

Facial lesions and symptoms were observed 4 and 12 weeks after the first treatment, and adverse reactions were recorded. The combination group showed significant improvement in symptoms 4 weeks after the first treatment, while the individual treatment groups showed no significant improvement. After three rounds of treatments, seborrheic dermatitis had significantly decreased in the three groups; the efficacy of the combined treatment group was significantly higher than that of the IPL group and the 30% supramolecular salicylic acid group.

**CONCLUSION:**

IPL combined with 30% supramolecular salicylic acid was effective in the treatment of facial seborrheic dermatitis and provided a quicker result with no adverse reactions.

## INTRODUCTION

Seborrheic dermatitis is a chronic inflammation that occurs at the base of the sebum spillage and usually manifests in the form of bright red or yellow patches with greasy scales or scabbed surfaces. It is often distributed in the zone rich in sebaceous glands. Patients involved in this study had this condition appear on the face, forehead, eye orbit, eyelid, and the nasolabial groove of the greasy erythema. The main clinical treatment is to dissolve the oil and cutin peeling, reduce inflammation, and relieve itching. The commonly used treatment methods are salicylic acid and intense pulsed light (IPL). However, the effect of a single treatment is slow, and the combined treatment can make up for the deficiency of single treatment. At present, the combination of salicylic acid and IPL in the treatment of seborrheic dermatitis has not been reported. We used IPL combined with 30% supramolecular salicylic acid in the treatment of facial seborrheic dermatitis and achieved satisfactory results.

## MATERIALS AND METHODS

### Participants

The clinical protocol of our study was approved by the Ethics Committee of Huamei Cosmetic Hospital, ethical code 2018(018). Before the experiment, the subjects were fully informed of the purpose, processes, possible risks, and benefits of our study, and all of them signed informed consent forms.

The participants were outpatients of the Department of Dermatology from September 2018 to September 2019. The inclusion criteria were the diagnosis of seborrheic dermatitis, which could be mild or moderate for those whose condition appeared on their faces, and patients who could complete the course. The exclusion criteria included patients who were allergic to salicylic acid, or received antibiotics, vitamin A, sex hormones, or glucocorticoids 3 weeks before treatment. Patients who used sun-exposure protection in the month prior to the study or had other face-related skin diseases were also omitted from this study. Furthermore, patients who were pregnant, lactated, had hyperlipidemia, or an abnormal liver or kidney function were also excluded.

### Cases of groups

A total of 45 patients with seborrheic dermatitis were included in this study, 13 were male and 32 were female aged 20-41 years with an average age of 28.4 years. They were randomly divided into three groups: the first group was the salicylic acid group, average age 28.8 years (20-40 years old), average treatment course of 15.4 months; the second group was the IPL group, average age 28.5 years (21-41 years old), average treatment course of 15.3 months; the third group was the IPL and salicylic acid group average age of 28.1 years (22-39 years old), average treatment course of 15.3 months. There were no significant differences in age or disease course and severity among the three groups.

### Instruments and parameters

Lumenis OneTM (Lumenis, San Jose, CA, USA), was used for the IPL treatment, and Boloda (Shanghai Ruizhi technology, Shanghai, China.) was used for the 30% supramolecular salicylic acid treatment.

### General instructions for patients

On the day of treatment, we carefully explained the post-operative precautions to the patient. We cleaned the skin thoroughly with facial cleansers and took photos. The groups and parameters of each treatment were recorded in detail once every 4 weeks; 3 times during the course of treatment. Patients were revisited to observe the symptoms 4 and 12 weeks after the first treatment; photos were taken, and the adverse reactions during treatment were recorded.

### Salicylic acid group

Patients were placed in the supine position avoiding the mouth, canthus, and nostrils while 30% supramolecular salicylic acid was applied to the forehead, nose, chin, and cheek for 3-8 minutes to determine whether to extend the residence time, depending on the skin and patient's tolerance. The endpoint of the treatment was determined by the degree of erythema and discomfort the subject experienced. Erythema reaction was considered either mild, moderate, or severe. If the erythema area <30%, it was considered mild; if the erythema was visible to the naked eye and the area of the erythema was between 30% and 60%, it was considered moderate, and the erythema was considered severe if the erythema area >60%. When the face appeared to have moderate erythema, we immediately wiped off the salicylic acid. Ice compression was used 15-30 minutes after treatment.

### IPL group

The patient was placed in the supine position and the laser-cold gel was applied. The corresponding filter, pulse number, pulse width, pulse delay, and energy density were set according to the skin color and skin lesion color of the patient. The filter was set at 560 or 590, while the pulse number, energy density, pulse width, and pulse delay were set at double or triple pulses, 12-14 j/cm^2^, 3.5-5.0 ms, and 30-35 ms, respectively. We repeated this once or twice. The result of the treatment was a reddish change in skin color that quickly subsided. After treatment, we applied a moisturizing mask and cold spray for 15-30 minutes.

### IPL and salicylic acid group

First, 30% supramolecular salicylic acid procedure was performed and treated with ice compression for 15-30 minutes. After the patient experienced no obvious burning or tingling sensation on the face, IPL therapy was performed.

### Efficacy evaluation index

The evaluation of the curative effect was combined with subjective evaluation and scoring. Two dermatologists evaluated the condition of the patient based on pre-operative and post-operative photographs and consultation. Each region was clinically evaluated for erythema, scale, and pruritus, and was given a score from 0 to 3 (0=clear, 1=mild, 2=moderate, and 3=severe) (Table 1). The index of curative effect = (total score before treatment-total score after treatment)/(total score before treatment)×100%. The index of curative effect ≥90% was recovery; excellence 60-89%; effective 20-59%; and invalid <20%. The percentage of recovery and excellence was the effective rate.

### Statistical analysis

The statistical package SPSS 19.0 (IBM Corp, NY, USA) was used to analyze the data; The Chi-square test was used to count the data. A *p*-value of <0.05 was considered significant.

## RESULTS

### Curative Effect

When 45 patients with seborrheic dermatitis were followed up 4 weeks after the first round of treatment, the symptoms had improved. The salicylic acid group showed 0 cases of excellence while 4 cases were considered effective; the effective rate was zero. The IPL group showed 0 cases of excellence while 5 cases were considered effective; the effective rate was zero. The salicylic acid and IPL group showed 8 cases of excellence while 4 cases were considered effective; the effective rate was 53.3%. The efficiency of the salicylic acid and IPL group was significantly higher than that of the salicylic acid group and IPL group respectively, and the difference of the three groups was statistically significant(*p*<0.05).

**Figure 1:**
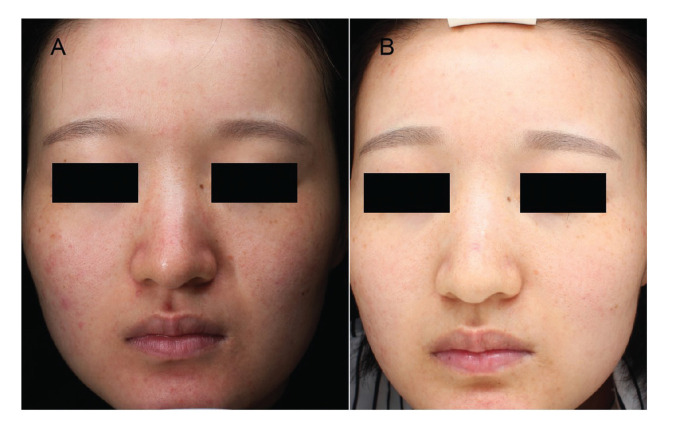
A 25-year-old female patient at baseline (A) and 4 weeks after the first treatment (B) of salicylic acid and intense pulsed light group. The red patches and erythema are significantly improved.

When the 45 patients diagnosed with seborrheic dermatitis were followed up 4 weeks after three rounds of treatment, the symptoms had improved. The salicylic acid group showed 9 cases of excellence while 2 cases were considered effective; the effective rate was 73.3%. The IPL group showed 8 cases of excellence while 3 cases were considered effective; the effective rate was 73.3%. The salicylic acid and IPL group showed 8 cases of excellence while 5 cases were considered effective; the effective rate was 86.7%. The efficiency of the salicylic acid and IPL group was significantly higher than that of the salicylic acid group and IPL group respectively, and the difference between the two groups was statistically significant (*p*<0.05) (Table 2) (Figure 1).

### Adverse reactions

No scar, hyperpigmentation, blisters, dermatitis, or other obvious adverse reactions were experienced by patients after treatment. Several patients in the salicylic acid group and combined treatment group desquamated and improved after the moisturizing treatment.

## DISCUSSION

Seborrheic dermatitis is an inflammatory skin disease that occurs in the sebum spillage, usually characterized by erythema and greasy scale (1,2). The pathogenesis of the disease is unclear. Current studies have shown that seborrheic dermatitis is closely related to the excessive sebum, the excessive reproduction of *Malassezia furfur* and *Staphylococcus epidermidis* (3,4), and the destruction of the skin barrier function (5). The application of antifungal drugs and glucocorticoids could alleviate the symptoms (6,7); however, long-term application will produce telangiectasia, epidermal atrophy, glucocorticoid dependence, and other adverse reactions (8,9). In recent years, it has been reported that salicylic acid (10,11) and IPL (12-15) have also been effective in treating seborrheic dermatitis. However, the efficacy and safety of IPL combined with salicylic acid in the treatment of seborrheic dermatitis has not been reported.

In this study, the effective rate of the salicylic acid group at week 4 was 0 while the effective rate at week 12 was 73.3%; This was also the case for the IPL group. The effective rate of the salicylic acid and IPL group was 53.3% at week 4 and 86.7% at week 12. Both the salicylic acid and IPL treatment alleviated the symptoms of seborrheic dermatitis, and the combined treatment group had a better and faster effect than the pure salicylic acid group and pure IPL group respectively. There was no significant difference between the pure salicylic acid group and the IPL group. Furthermore, there were no obvious adverse reactions recorded in the three groups.

In 1928, researchers extracted salicylic acid, also known as doxy acid, from willow barks. In the field of dermatology, salicylic acid is an oil-soluble organic acid with anti-inflammatory and broad-spectrum antibacterial properties (16). Supramolecular salicylic acid is a controlled, sustained-release salicylic acid (17), which gives high transdermal efficiency and good tolerance. Salicylic acid can dissolve oil plugs, inhibit the oil secreted by sebaceous gland cells, and inhibit the excessive proliferation of sebaceous gland cells which could act on the pathogenesis of seborrheic dermatitis. Salicylic acid has an inhibitory effect on *Malassezia furfur* and *Staphylococcus epidermidis* caused by seborrheic dermatitis. Salicylic acid can also improve the erythema of seborrheic dermatitis. Moreover, for patients with seborrheic dermatitis with abnormal skin barrier functions, the new supramolecular salicylic acid was milder, with fewer side effects, and could be tolerated for an extended amount of time.

After IPL irradiation on the dermis, type I and type III collagen in the dermis increased; the infiltration of dermal inflammatory cells subsided, and the epidermis thickened (18). The IPL could stimulate the defense function of the tissue leading to bacteriostasis. Some scholars believe that the photothermal therapy of IPL can activate porphyrin to release the oxygen ions of singlet (or free state); the monomorphic oxygen would kill the chaff *Malassezia furfur* and *Staphylococcus epidermidis*, thus limiting the growth of bacteria (19,20). At the same time, IPL could reduce, or even close the capillaries connected to the sebaceous glands, thus reducing or inhibiting the secretion of the sebaceous glands. This provided a theoretical basis for the treatment of seborrheic dermatitis by IPL. In this study, we applied IPL to treat seborrheic dermatitis, and after 3 full courses of treatment, a good effect was achieved.

In this study the patients with seborrheic dermatitis were accompanied by skin barrier damage, thus single salicylic acid or IPL were relatively mild, making the onset time of single treatment slow with symptoms only being controlled after a course of treatment. Salicylic acid combined with IPL therapy compensated for this deficiency resulting in a significant improvement with no increased risk of complications after a single treatment. It was speculated that it was related to the synergistic effect of the IPL and salicylic acid. IPL could make up for the lack of shallow penetration depth of the salicylic acid, while salicylic acid could enhance the anti-inflammatory and oil-control effect of the IPL, thus contributing to the rapid and safe control of symptoms.

## CONCLUSIONS

To conclude, IPL combined with 30% supramolecular salicylic acid is a quick and effective treatment for seborrheic dermatitis with less adverse reactions.

## AUTHOR CONTRIBUTIONS

Gu RL and Wang SQ participated in the design of this study and all performed the statistical analysis. Wang SQ carried out the study, collected important background information and drafted the manuscript. All authors read and approved the final manuscript.

## Figures and Tables

**Table 1:** Scoresheet of Symptoms and Signs of Seborrheic Dermatitis.

	0 pts	1 pts	2 pts	3 pts
Erythema	Clear	Reddish	Pale red	Red
Scale	Clear	A little	Medium	Obvious
Pruritus	Clear	Mild	Moderate	Severe

pts: primary traits scales.

**Table 2:** Effective rate after treatment of each group.

	Very satisfied or satisfied			
Variable	Effective rate after 4 weeks	Effective rate after 12 weeks	χ^2^	*p*
Salicylic acid group	0	73.3%		
IPL group	0	73.3%		
Salicylic acid and IPL group	53.3%	86.7%	68.39	*p*<0.0001

IPL: intense pulsed light.
